# Molecular characterization of HTLV-1 gp46 glycoprotein from health carriers and HAM/TSP infected individuals

**DOI:** 10.1186/1743-422X-10-75

**Published:** 2013-03-06

**Authors:** Aline C A Mota-Miranda, Fernanda K Barreto, Maria F C Amarante, Everton Batista, Joana P Monteiro-Cunha, Lourdes Farre, Bernardo Galvão-Castro, Luiz C J Alcantara

**Affiliations:** 1 Centro de Pesquisa Gonçalo Moniz, Fundação Oswaldo Cruz, CPqGM/FIOCRUZ. Rua Waldemar Falcão 121, Brotas, Salvador, Bahia, 40295-001, Brazil; 2 Escola Bahiana de Medicina e Saúde Pública, Salvador, Bahia, Brazil; 3 Universidade Federal da Bahia, Instituto de Ciências da Saúde, Salvador, Bahia, Brazil; 4 Laboratório de Patologia Experimental, Centro de Pesquisa Gonçalo Moniz, Fundação Oswaldo Cruz, Salvador, Bahia, Brazil; 5 National Cancer Institute, National Institutes of Health, Bethesda, MD, USA

**Keywords:** HTLV-1, HAM/TSP, Gp46, Mutation

## Abstract

**Background:**

Human T-cell Leukemia Virus type 1 (HTLV-1) is the etiological agent of tropical spastic paraparesis/HTLV-associated myelopathy (HAM/TSP) that can be identified in around 0.25%–3.8% of the infected population. Disease progression can be monitored by the proviral load and may depend on genetic factors, however, it is not well understood why some HTLV-1 infected people develop the disease while others do not. The present study attempts to assess the molecular diversity of gp46 glycoprotein in HAM/TSP patients and Health Carrier (HC) individuals.

**Methods:**

Blood samples were collected from 10 individuals, and DNA was extracted from PBMCs to measure the HTLV-1 proviral load. The gp46 coding sequences were amplified PCR, cloned and sequenced. The molecular characterization was performed using bioinformatics tools.

**Results:**

The median HTLV-1 proviral load of HC (n = 5) and HAM/TSP (n = 5) patients was similar (average 316,227 copies/10^6^ PBMCs). The gp46 molecular characterization of 146 clones (70 HC and 76 HAM/TSP) revealed an overall diversity, within HC and HAM/TSP clones, of 0.4% and 0.6%, respectively. Five frequent mutations were detected among groups (HAM/TSP and HC clone sequences). A single amino acid (aa) substitution (S35L) was exclusive for the HC group, and three gp46 substitutions (F14S, N42H, G72S) were exclusive for the HAM/TSP group. The remaining frequent mutation (V247I) was present in both groups (p = 0.0014). The in silico protein analysis revealed that the mutated alleles F14S and N42H represent more hydrophilic and flexible protein domains that are likely to be less antigenic. The Receptor Binding Domain is quite variable in the HAM/TSP group. Two other domains (aa 53–75 and 175–209) that contain multiple linear T-cell epitopes showed genetic diversity in both HAM/TSP and HC groups. Further analysis revealed 27 and 13 T-cell epitopes for class I HLA alleles and class II HLA alleles, when analyzing the entire gp46.

**Conclusions:**

The most common gp46 mutations were not associated clinical status because they were found in only one individual, except for the V247I mutation, that was found at viral clones from HAM/TSP ad HC individuals. Because of this, we cannot associate any of the gp46 found mutations with the clinical profile.

## Background

HTLV-1 was initially isolated from a patient with cutaneous T-cell lymphoma in early 1980s, being the first discovered human retrovirus [1]. HTLV-1 is classically the etiological agent of tropical spastic paraparesis/HTLV-associated myelopathy (HAM/TSP), which is characterized, besides the spastic paraparesis, by urinary disturbances and, in almost all patients, spasticity and/or hyper-reflexia of the lower extremities [2]. HTLV-1 has also been implicated as the cause of Adult T-cell Leukemia/Lymphoma (ATLL) and of other inflammatory disorders such as arthritis, uveitis, dermatitis, lymphadenitis and Sjogren’s syndrome [2].

It is estimated that around 0.25%–3.8% of the HTLV-1 infected population develops HAM/TSP [3-6]. Despite the scarcity of data on the real prevalence of HAM/TSP in the Brazilian population, the individual risk of disease development is of considerable importance for HTLV-1 endemic areas such as southern Japan, the Caribbean, Central and South America (including Brazil), the Middle East, Melanesia, and equatorial regions of Africa [4]. The progression to HAM/TSP includes viral factors such as proviral load and host genetic factors such as Human Leukocyte Antigen (HLA). Most clinical studies associate proviral load to increased risk of disease progression, and in some of them, the HTLV-1 proviral load progressively increases according to HAM/TSP ascertainment level (possible, probable, and definite) of the De Castro-Costa diagnostic criteria [7,8]. On the other side, molecular data from southern Japan indicates that the alleles HLA-A*02 and Cw*08 (HLA class I) are associated with a significant reduction in both HTLV-1 proviral load and the risk of HAM/TSP, whereas HLA-B*5401 (HLA class I) and HLA-DRB1*0101 (HLA class II) predispose to HAM/TSP in the same population [9,10].

Although most studies of HTLV-1 genotype have reported no association between HTLV-1 variants and risk to HAM/TSP, it is known that HAM/TSP patients generally have higher anti-HTLV-1 antibody titer than Health Carriers (HC) with a similar proviral load [11]. Additionally, the antibody reactivity to Tax epitopes can be different between HAM/TSP (71%–93%) and HC (27%–37%) individuals [12]. Regarding the regulatory protein genotypes, a p12 K88 allele was found mainly among HAM/TSP individuals, suggesting that it could be implicated as a pathogenesis marker [13].

The HTLV-1 envelope glycoproteins (Env) mediate the binding of the virus to its receptor on the surface of target cells and the subsequent fusion of virus and cell membranes. It is also important to note that three different molecules are assumed to participate in HTLV-1 entry: a glucose transporter type 1 (GLUT-1), the VEGF-165 receptor Neuropilin 1 (NRP-1), and heparan sulfate proteoglycans (HSPG) [14-16]. Throughout the characterization of neutralizing antibodies and analysis of Env mutants, it is now known that gp46 contains a great number of functional and immunodominant domains that are associated to the prevalence of linear epitopes, interaction to neurophilin 1 or heparan sulfate molecules, and induction of antibodies [13,17-20].

Because it is not well understood why some HTLV-1 infected people develop disease while others do not, the purpose of the present study was to assess the molecular diversity of gp46 glycoprotein in HAM/TSP and HC individuals to identify the potential molecular biomarkers and/or the therapeutic targets for HAM/TSP. Despite the lack of association with the clinical status, five frequent mutations at gp46 were identified.

## Results

### Sample characterization

**Figure 1:**
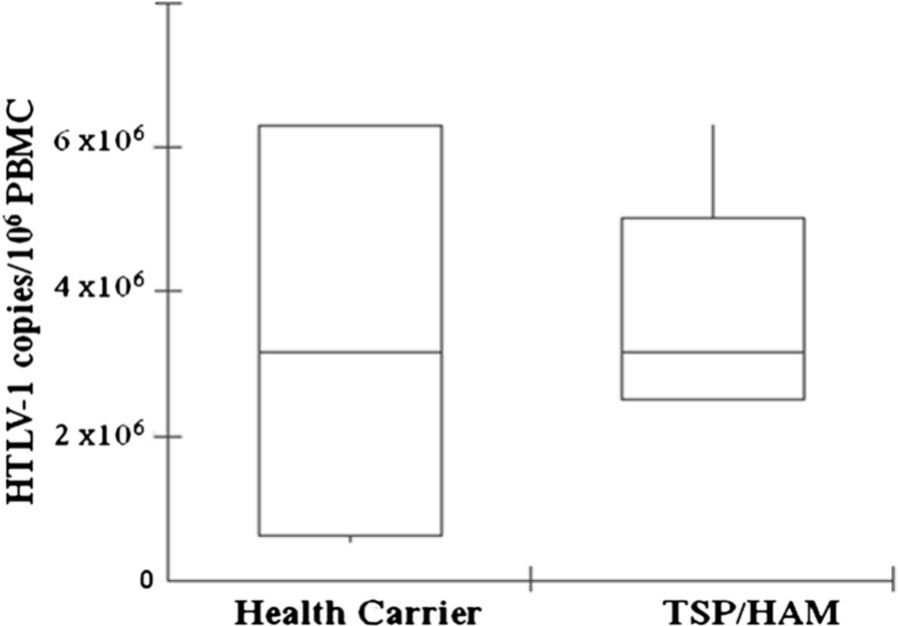
**Median of HTLV-1 proviral load in health carriers (n = 5) and HAM/TSP (n = 5) individuals.** Results are presented as number of HTLV-1 copies/10^6^ PBMCs. The box-plots represent the median, interquartile range (boxes) and the 5–95% data range (whisker caps).

Blood samples were collected from 10 HTLV-1 infected individuals recruited from a reference health unit: 5 HC and 5 HAM/TSP patients. Because the progression to HAM/TSP is multifactorial and is not only dependent on viral factors, but also on the host’s genetic background, we choose all individuals with similar proviral load and age. The median HTLV-1 proviral load of HC and HAM/TSP patients was the same (316,227 copies/10^6^ PBMCs) (Fisher Test, *p* = 0.0007-after Bonferroni Correction) (Figure 1), while the median age was 53 (38–72) and 58 (41–73) years for HC and HAM/TSP patients, respectively (*p* = 0.239).

### Molecular characterization of gp46 glycoprotein

**Figure 2:**
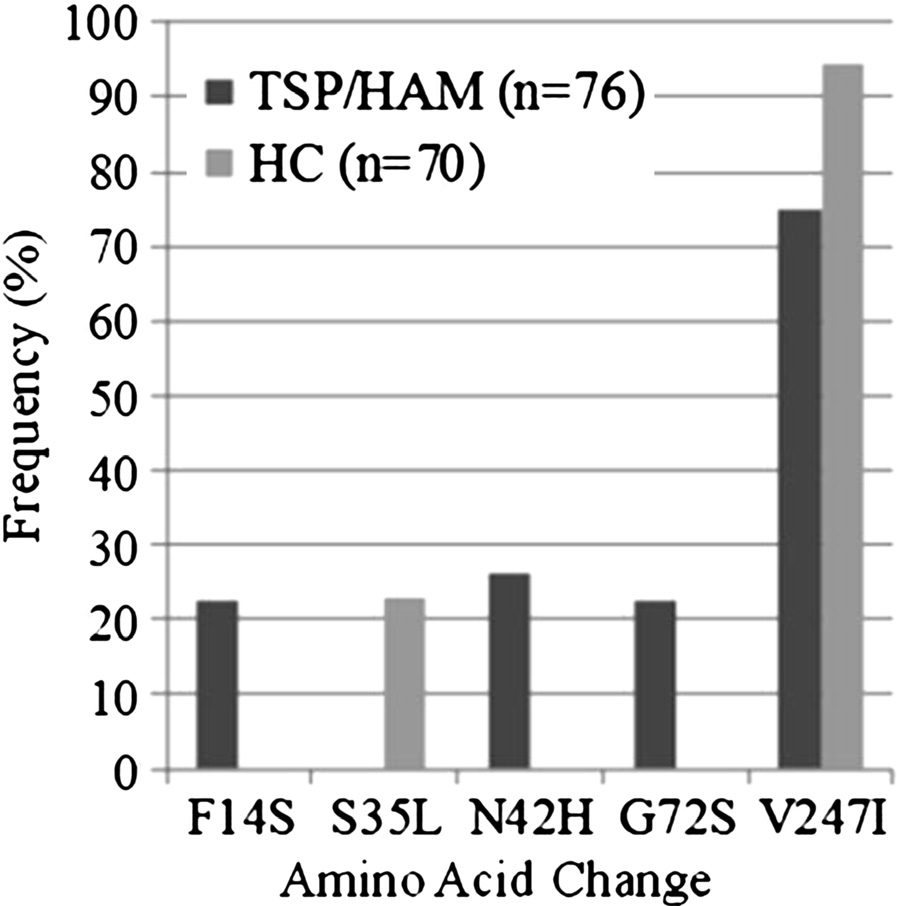
**Frequency (%) of the most common amino acid changes at gp46 clone sequences from HAM/TSP infected individuals and Health Carriers (HC).** N is the number of clone sequences.

We obtained 146 gp46 clone sequences (908pb, 302aa): 70 from HC (average of 14 clones per individual) and 76 from HAM/TSP individuals (average of 15 clones per individual). The HC and HAM/TSP clone sequence overall diversity was 0.4% and 0.6%, respectively. Regarding the amino acid changes, we identified five mutations with frequency over 20% within the clone groups (Figure 2). Among these common mutations, one (S35L) was exclusive to the HC group, three (F14S, N42H, G72S) were exclusive to the HAM/TSP group and, finally, one mutation (V247I) was found in both groups but with a statistically significant difference in the frequency within the groups (Fisher Test, *p* = 0.0014).

Among the five mutations found, three (F14S, S35L and N42H) were not present in any of the 42 Brazilian sequences used to create the consensus. G72S was found in one *Brazilian* sequence; however, we do not have information about the geographic region from which the sequence was obtained. Finally, the V247I mutation was found in sequences from all different geographic regions represented in our dataset. Similarly, F14S, S35L, N42H and V247I mutations were not present in any sequence of the *Cosmopolita dataset,* while G72S mutation was found in sequences from Gabon, Martinique, French Guyana and Guadalupe.

**Figure 3:**
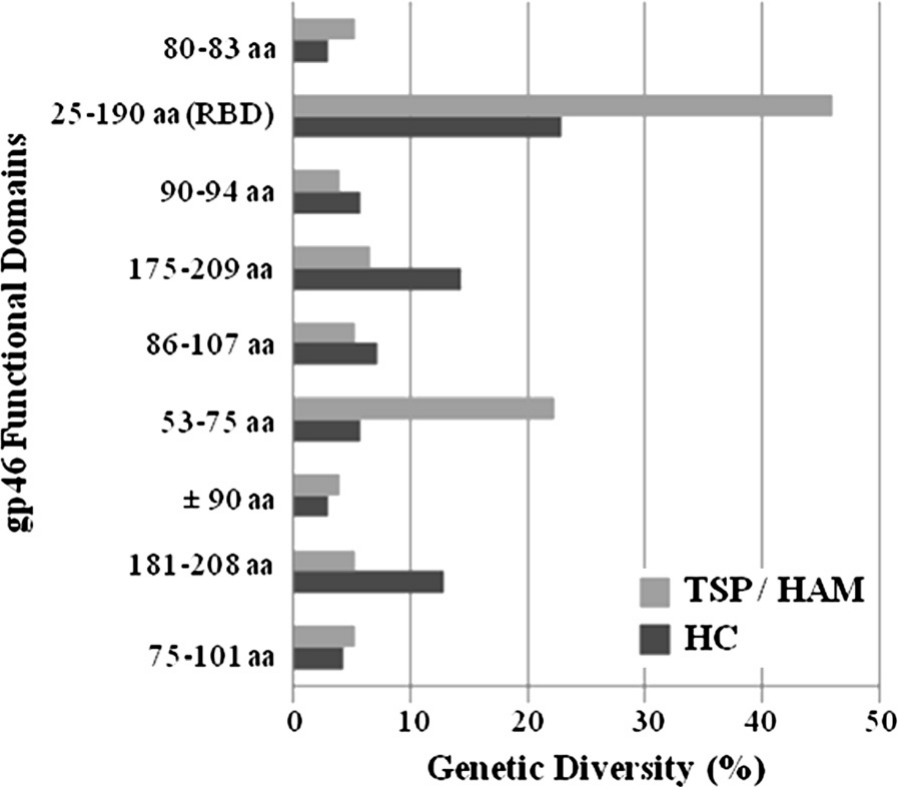
**Genetic diversity of HTLV-1 gp46 functional domains in Health Carrier (HC) and HAM/TSP clone sequences.** aa means amino acid.

We used the already known gp46 functional domains to perform the search for mutations (See Additional file 1). In general, the Receptor Binding Domain (RBD) presented the greatest number of amino acid changes in both groups. However, the general diversity (frequent and not frequent mutations) profile is different between the HC and HAM/TSP groups. The HC group presented the three most divergent domains, and one of them is also a divergent domain in the HAM/TSP group (Figure 3). It is interesting to note that the second most divergent domain (175-209 aa) in the HC group is characterized by the prevalence of linear epitopes, and it was not so divergent in the HAM/TSP group. It was also noticeable that the domain 53-75 aa, associated to the most prevalent occurrence of linear epitopes, had the second higher index of divergence among the HAM/TSP group (Figure 3). This domain is less divergent in the HC group of clones. The 197 amino acid site, which was already associated to the decrease in cellular fusion, was mutated (D197N) in two clone sequences in our data set: one from the HC group and the other from the HAM/TSP group (data not shown). Moreover, one HAM/TSP sequence was mutated (Y80C) at position 80 aa, which is part of the YSLY motif involved in the protein trafficking to the plasma membrane.

We also performed the epitope prediction in the domains with the higher genetic diversity in HAM/TSP (53-75 aa) and HC clones (175-209 aa). Analyzing the 175-209 aa domain, 16 HLA-I and seven HLA-II specific epitopes were found (Table 1). On the other hand, 11 HLA-I and five HLA-II specific epitopes were identified in the 53-75 aa domain (Table 2). Since the G72S mutation is located in the 53-75 aa domain, we tested both wild type and mutated alleles. The mutated allele was associated with change in HLA binding score in three of the 11 epitopes including this position (72 aa). For the HLA A26 and A01 (HLA-I) specific epitopes, starting at positions 65 aa and 68 aa, the HLA binding score increased and decreased, respectively (Table 2). For the HLA DRB1 0101 (HLA-II) specific epitope, starting at position 64 aa, the HLA binding score decreased (Table 2).

**Table 1:** Possible epitopes for classes I and II HLA alleles in HTLV-1 gp46 175-209 aa domain

**A-Class I HLA alleles**																
**AA position**	**Epitope sequence**															**HLA allele**
175	F	L	N	T	E	P	S	Q	L	A 0201, B 08						
177	N	T	E	P	S	Q	L	P	P	A 01, A 1101, B 4501						
179	E	P	S	Q	L	P	P	T	A	A 0702						
182	Q	L	P	P	T	A	P	P	L	A 0201, B 08, B 4402						
183	L	P	P	T	A	P	P	L	L	A 0701, B 08, B 5101						
184	P	P	T	A	P	P	L	L	P	B 0702						
185	P	T	A	P	P	L	L	P	H	A 1101						
188	P	P	L	L	P	H	S	N	L	B 0702, B 02, B 1402, B 2705, B 37, B 5101						
191	L	P	H	S	N	L	D	H	I	B 4901, B 5101						
192	P	H	S	N	L	D	H	I	L	B 1510, B 3801 B 3901						
193	H	S	N	L	D	H	I	L	E	A 1101						
194	S	N	L	D	H	I	L	E	P	B 4501						
199	I	L	E	P	S	I	P	W	K	A 03, A 1101, A 6801, B 2705						
202	P	S	I	P	W	K	S	K	L	A 26, B 2705						
203	S	I	P	W	K	S	K	L	L	A 0201, B 08, B 37						
205	P	W	K	S	K	L	L	T	L	A 26, B 3901						
**B-Class II HLA alleles**																
**AA position**	**Epitope sequence**															**HLA allele**
172	P	I	W	F	L	N	T	E	P	S	Q	L	P	P	T	DRB 0701
180	P	S	Q	L	P	P	I	A	P	P	L	L	P	H	S	DRB 0301, DRB 0701
187	A	P	P	L	L	P	H	S	N	L	D	H	I	L	E	DRB 0301, DRB 1501
188	P	P	L	L	P	H	S	N	L	D	H	I	L	E	P	DRB 0701
193	H	S	N	L	D	H	I	L	E	P	S	I	P	W	K	DRB 0701
194	S	N	L	D	H	I	L	E	P	S	I	P	W	K	S	DRB1 0101
201	E	P	S	I	P	W	K	S	K	L	L	T	L	V	Q	DRB 1501

**Table 2:** Possible epitopes for classes I and II HLA alleles in HTLV-1 gp46 53-75 aa domain

**A-Class I HLA alleles**																
**AA position**	**Epitope sequence**															**HLA allele**
52	L	D	L	L	A	L	S	A	D	B 37						
53	D	L	L	A	L	S	A	D	Q	A 03						
54	L	L	A	L	S	A	D	Q	A	A 0201, A 03						
55	L	A	L	S	A	D	Q	A	L	A 0201, B 08, B 1402, B 5101						
56	A	L	S	A	D	Q	A	L	Q	A 03						
57	L	S	A	D	Q	A	L	Q	P	A 1101						
62	A	L	Q	P	P	C	P	N	L	A 0702, B 08, B 2705, B4402						
63	L	Q	P	P	C	P	N	L	V	B 3902						
64	Q	P	P	C	P	N	L	V	G	B 0702, B 5101						
65	P	P	C	P	N	L	V	G	Y	A 01, A 26, B 4402						
68	P	N	L	V	G	Y	S	S	Y	A 01						
**B-Class II HLA alleles**																
**AA position**	**Epitope sequence**															**HLA allele**
51	T	L	D	L	L	A	L	S	A	D	Q	A	L	Q	P	DRB1 0101, DRB 0401
52	L	D	L	L	A	L	S	A	D	Q	A	L	Q	P	P	DRB 0301, DRB 0701
54	L	L	A	L	S	A	D	Q	A	L	Q	P	P	C	P	DRB1 0101, DRB 07071, DRB 1501
64	Q	P	P	C	P	N	L	V	G	Y	S	S	Y	H	A	DRB1 0101
67	C	P	N	L	V	G	Y	S	S	Y	H	A	T	Y	S	DRB1 0101, DRB 0401, DRB 0701, DRB 1501

To identify if the amino acid mutations found has been positively selected, we have performed positive selective pressure analysis. According to our results, the ω value calculated for the clone sequences from HAM/TSP individuals was higher than the ω value calculated for the clone sequences from HC individuals. Despite this result no calculated ω value was >1.

The identification of post-translation modification sites is summarized in Table 3 and shows that the same potential protein domains were found among the HC and HAM/TSP groups of sequences. However, despite being the same sites, their frequencies were statistically different, being more frequent among the viral clones isolated from HAM/TSP individuals (Table 3).

**Table 3:** Potential protein domains in HTLV-1 gp46 from health carriers and HAM/TSP individuals

**Potential protein domain**	**Health carrier**		**HAM/TSP**	
	**(N = 70)**		**(N = 76)**	
	**Amino acid site**	**Frequency (%)**	**Amino acid site**	**Frequency (%)**
N-Myristilation site	97-102	91.4	97-102	100^*^
CK2-phosphorylation site	103-106	91.4	103-106	98.7^§^
	194-197	88.6	194-197	100^¶^
N-glycosilation	140-143	90	140-143	98.7^Φ^
	222-225	91.4	222-225	100^†^
	244-247	91.4	244-247	98.7^‡^
	272-275	92.8	272-275	98.7^≠^
PKC-phosphorylation site	109-111	91.4	109-111	100^Δ^

The numbers superscript at the last column represents the results from the Exact Fisher Test to the frequency of the potential protein domains in the two different groups. ^*^p = 0.028, ^§^*p* = 0.0551, ^¶^*p* = 0.0022, ^Φ^*p* = 0.0283, ^†^*p* = 0.0108, ^‡^*p* = 0.0551, ^≠^*p* = 0.1047, ^Δ^*p* = 0.0108. N means the number of clones.

**Figure 4:**
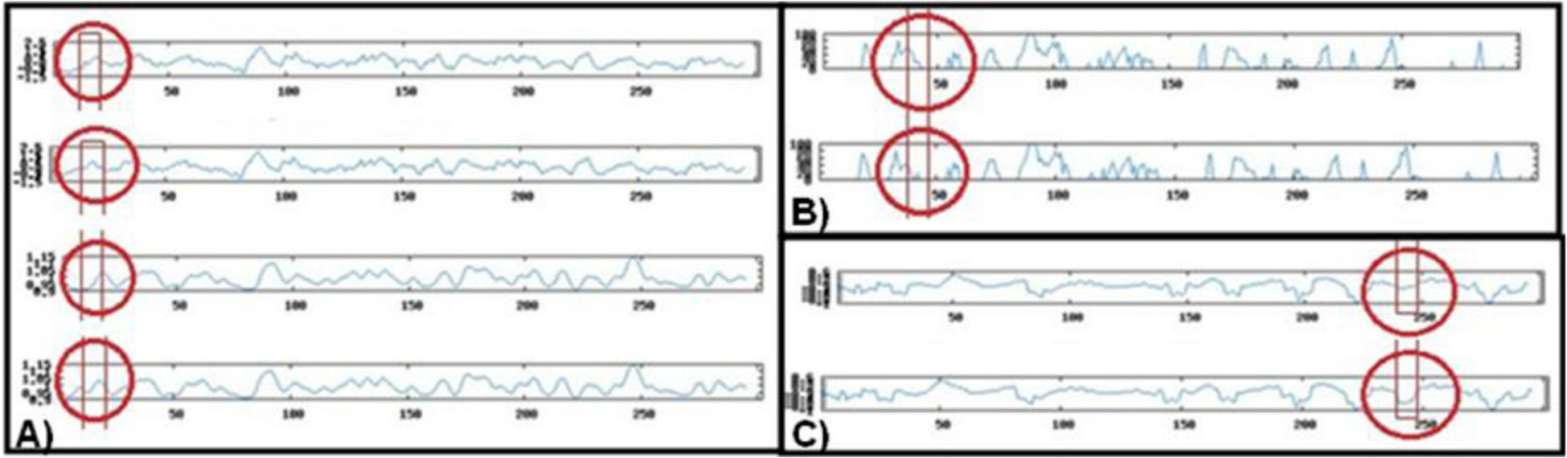
**Physico-chemical analysis of wild type and mutated alleles of F14S, N42H and V247I polymorphisms.** In Figure 4**A** (F14S), the graphs are organized as follows: hydrophilicity for wild type allele, hydrophilicity for mutated allele, flexibility for wild type allele, and flexibility for the mutated allele. In Figure 4**B** (N42H) and 4**C** (V247I), the graphs are organized as follows: antigenicity for wild type allele and antigenicity for mutated allele.

We submitted wild type and mutated sequences to physico-chemical analysis using the NPSA tool. The following was observed: 1) the mutated allele presenting the F14S mutation was more hydrophilic and flexible than the wild type allele (Figure 4A); 2) the mutated allele presenting the N42H mutation was less antigenic than the wild type allele (Figure 4B); 3) the mutated allele presenting the V247I mutation was less antigenic than the wild type allele (Figure 4C). According to our results, other less frequent mutations were associated to changes in the physico-chemical profile and potential protein domains: the T142I mutation was associated to the loss of a N-glycosylation site and to a decrease in antigenicity; the F159S mutation presented a more hydrophilic and flexible profile, associated to the insertion of a N-Myristilation site; and the H290D, C289R and F141S mutations were characterized by an increase in hydrophilicity and flexibility, the opposite of the D197N mutation which was associated to a decrease in flexibility (Data not shown).

**Figure 5:**
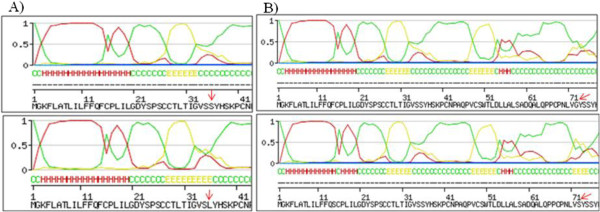
**HTLV-1 gp46 secondary structure prediction.** The S35L (**A**) and G72S (**B**) polymorphisms were associated to the switch of coil to extended beta structure.

After submission of wild type and mutated sequences to SWISS-MODEL server, the results showed that the S35L and G72S mutations were associated to the change of coil for extended beta at the secondary structure prediction (Figure 5).

## Discussion

The low overall genetic diversity found in gp46 sequences is in agreement with the fact that the HTLV-1 genome exhibits relatively few sequence variations, emphasizing that recombinant envelope glycoproteins or synthetic peptides could be used as an effective vaccine candidate. This genomic stability and the presence of neutralizing antibodies in HTLV-1-infected individuals are favorable factors for the design of an efficient HTLV-1 vaccine, and this could be an advantage comparing to the challenge of developing a vaccine to HIV infection. However, it is important to note that the sequences from HAM/TSP individuals are relatively more divergent compared to those from HC individuals, confirming that the immune environment represents effective selective pressure.

It is also worth noting that the relative lack of variation in the HTLV gp46 relative to the HIV gp120 is believed to reflect the fact that HTLV-1 persists in an individual by clonal expansion of HTLV-1-infected cells, rather than continual spread of the virus, as in HIV infection. However, there are minor sequence variations between geographical regions and certain HTLV-1 subgroups that can be associated with different risks of HAM/TSP. Furthermore, a quarter of the provirus load may be carried by CD8+ T cells and when equilibrium is reached between a persistently replicating pathogen and the immune response, the frequency of specific CD8+ T cells is an unreliable index of the efficiency or effectiveness of the T cell response [21].

We identified five common mutations in all 176 gp46 sequences. Four of the most common mutations (except the V247I mutation) were associated to an intra-host profile because were specifically found in the same single individual: the S35L mutation was exclusive for one HC individual, the F14S and G72S mutations were exclusive for one HAM/TSP individual, and finally the N42H change was exclusive for other HAM/TSP individual. Because of this, we cannot associate any of these mutations to the clinical profile, due to the reduced number of individuals recruited in this study. Due to the observed intra-host profile of the found mutations, we compared them to the proviral load of each individual. The mutations S35L, F14L, G72S and N42H were identified in individuals with high proviral load (log > 5).

We suggest that future studies evaluating the molecular diversity of envelope proteins should take into account a larger number of individuals, and a reduced number of clones. A larger number of individuals could be useful to evaluate the association of the possible mutations to the clinical status, while cloning could be useful for the identification of intra-host variations and possible emerging changes. In this regard, three (F14S, S35L and N42H) of these newly identified mutations can be classified as a completely new protein characteristics, while the V247I mutation is an already known characteristic of Brazilian HTLV-1 isolates.

We could identify that HTLV-1 gp46 from HC and HAM/TSP individuals had different genetic diversity profiles, especially in domains with prevalence of linear epitopes. In these domains, we identified different epitopes between HC and HAM/TSP individuals. Recent data revealed that patients with HAM/TSP recognized a significantly broader repertoire of Env epitopes, compared with health carriers, suggesting that the diversity, frequency and repertoire of HTLV-1 Env CD8^+^ T-cells may be related to the hyperimmune response in HAM/TSP disease [22]. Additionally, the identification of the Env epitopes associated with the immune system activation could be useful to vaccine design [23]. In a recent report, the immunogenicity and protective efficacy in squirrel monkeys of a B-cell env gene epitope and of a T-cell multiepitope derived from Tri-Tax were tested. The authors showed that the reduction in the number of cells producing IFN-c in response to the Tri-Tax T-cell epitope peptide was much lower than with the B-cell env gene epitope peptide, indicating the involvement of other virus-specific T-cell subsets in this cellular immune response [24]. These T-cell subsets could be those ones that are specific to divergent viruses.

We believe in the potential of these mutations to become clinical markers as they are located in important protein domains. The fact that some of these mutations have been associated to physico-chemical or secondary structure changes does not mean that there is no need for *in vitro* assays. This investigation is important because antibodies are most likely to recognize linear epitopes but they are also dependent on the conformation of the antigen and efficient epitope recognition determines the neutralizing properties of anti-HTLV-1 antibodies [25]. In this case, non effective antibodies could be closely related to the clinical manifestation because they can play an important role in controlling HTLV-1 dissemination and the viral burden on natural infections, in particular through antibody-dependent cellular cytotoxicity [26].

The genomic regions where the post-translational sites were identified, showed a high identity among the sequences, suggesting the influence of these sites on the host immune response, and virus latency. The post-translational modifications in envelope glycoproteins are not well documented therefore there is a lack of evidence of the importance of these sites for virus clonal expansion. However, these sites are frequently found in HTLV-1 envelope proteins, and it would be interesting to investigate the functional impact of them. Regarding the Tax protein, for example, much is known about the post-translational modifications and they are important in the constitutive activation of NF-κB pathways, inhibition of DNA repair, activation of p53 tumor suppressor and cell cycle control [27].

The immune responses that differentiate health carrier from HAM/TSP could be a result of the fact that infected cells, which display the envelope on the surface, remain insensitive and not vulnerable to antibody-dependent cellular cytotoxicity and antibody-mediated complement fixation. From the present results, our group is carrying out a site-directed mutagenesis assay to test the impact of the described mutations in the binding capacity of the variant protein against natural antibodies produced by HC and HAM/TSP individuals. The benefits of investigating these mutations can also be useful for the Western Blot indeterminate profile which is dependent on the efficient binding of gp46 and natural antibodies.

## Conclusions

In conclusion, the results presented in this study suggest that the most frequent gp46 mutations (F14S, N42H, V247I and S35L), as well as, the mutations (H290D, C289R, F141S, D197N and F159S) associated to the loss of a post-translational site should be evaluated in greater detail, in particular the reactivity of the mutated proteins against natural antibodies. Furthermore, we believe that analyses involving a greater number of sequences can provide more information about the occurrence of these variations and the identification of others, which are just as important as the ones presented here.

## Methods

### Patients

Blood samples were collected from 10 individuals who were frequently seen at the Bahia School of Medicine and Public Health, HTLV reference center in Salvador, located in Northeastern Brazil, in the first period of 2010. This is a free public outpatient clinic that has provided comprehensive care to a total of 1,050 patients since 2002. All 10 patients were included in the present study with inclusion criteria consisting of an available HTLV-1 proviral load measurement and a neurological evaluation. All patients had a quite similar proviral load and median of age. All cases of co-infection were excluded, as well as symptomatic patients with other HTLV-1-associated diseases, such as infective dermatitis, uveitis, ATLL, Sicca syndrome, etc. The Disability Status Scale (DSS) [28] and the Osame Motor Disability Score (OMDS) were regularly applied by a neurologist. This study was approved by the Ethical Committee (Protocol Number 81/2007) of the Bahia School of Medicine and Public Health. Informed consent was obtained from all enrolled patients. Salvador is the capital of Bahia State in northeast Brazil and presents wide socioeconomic differences. The population is roughly 80% black or racially mixed African and Portuguese descendents.

### Measurement of HTLV-1 proviral load

PBMCs were obtained from EDTA blood by density gradient centrifugation. DNA was extracted using spin column DNA extraction system (Qiagen, Hilden, Germany) and HTLV-1 proviral load was quantified using a real-time TaqMan Polymerase Chain Reaction (PCR) method, as described previously [29]. Albumin DNA was used as an endogenous reference. The value of HTLV-1 proviral load was reported as the [(HTLV-1 average copy number)/(albumin average copy number)] × 2 × 10^6^ and expressed as the number of HTLV-1 copies per 10^6^ cells in PBMCs.

### gp46 PCR and cloning

The gp46 coding sequences from the viruses isolated from the PBMCs of HTLV-1-infected patients were amplified by PCR. In brief, gp46F/gp46R (gp46F 5^′^CGCCGATCCCAAAGAAAAA3^′^/gp46R 5^′^ACATGGAGCCGGTAATCCC3^′^) were designed and used to amplify a 936pb fragment, corresponding to the entire gp46 coding region. The PCR products were purified using the Qiaquick Gel Extraction kit (Qiagen, QIAamp DNA miniKit, Qiagen, Hilden, Düsseldorf, Germany). The amplicons were then ligated into the pCR®4-TOPO available at TOPO TA cloning® kit for sequencing (Invitrogen, CA, USA), and the mini preparations were made using the kit (Wizard® Plus Minipreps DNA Purification System). All purified clones were sequenced in an ABI Prism 3100 DNA Sequencer (Applied Biosystems Inc., Foster City, CA) using Taq FS Dye (Applied Biosystems) terminator cycle sequencing. Besides the same PCR primers, a new inner primer (gp46 R2 5^′^GACGTGCCAAGTGGATAGGC3^′^) was used to optimize the sequencing reactions.

### gp46 Molecular characterization

Before analyzing the obtained gp46 sequences for the presence of mutations and identification of molecular characteristics, two datasets were constructed. The first was called *“Cosmopolita database”* and was composed by 27 gp46 sequences from different countries (except from Brazil), previously available in the NCBI/Nucleotide Sequence Database (GenBank). It is important to note that the 27 sequences of the *“Cosmopolita dataset”* did not have any information about subtype and were generated from different countries: French Guyana, Central America (n = 7); Martinique, Central America (n = 1); Gabon, Central Africa (n = 12); and Guadalupe, Central America (n = 7). The second dataset was called *“Brazilian dataset”* and was composed by 42 gp46 sequences from Brazil, previously available in GenBank. It is therefore important to note that these 42 Brazilian sequences are classified as subtype a (Cosmopolita), and were generated from different Brazilian geographic regions: Salvador, Northern (n = 21); Londrina, South (n = 5); São Paulo, Southeast (n = 11), while five of them did not present any information about their geographic origin.

Both datasets were submitted separately to the Clustal X software [30] to perform the alignment which was then manually edited using the GeneDoc program [31], and finally, the edited alignments were used to generate a unique consensus sequence of each dataset using Bioedit software [32]. The consensus sequence from *“Cosmopolita database”* was called *“Cosmopolita reference”* and the consensus sequence from the *“Brazilian dataset”* was called *“Brazilian reference”*. These consensus sequences comprise the most frequent nucleotide variants found in previously published gp46 sequences from Brazil and elsewhere. These consensus sequences were used as the reference sequences to identify possible mutations in the 146 newly generated gp46 sequences.

The genetic distances were measured within the two distinct groups: gp46 sequences from HC and HAM/TSP HTLV-1 infected individuals. The Tamura Nei model was used with a distance matrix implemented in the MEGA 3.0 package [33], and the standard error computation was obtained by Bootstrap analysis (1000 replicates). The mutation/polymorphism identification was performed manually using the visualization of alignment in the Bioedit software.

To test the hypothesis that the amino acid substitutions within the gp46 sequences could have been favored or not by natural selection, the positive selection was assessed using six different codon-based maximum-likelihood substitution models [34]. All models were implemented in the HYPHY program [35] and the ω and *p* values were estimated through maximum-likelihood optimization, in such a way that using the M3 model, sites with a posterior probability exceeding 90% and a ω value > 1 were labeled as being “positive selection sites”. Finally, Likelihood Ratio Test (LRT) analysis was used to determine: (1) if site heterogeneity selection was present and (2) if there were positively selected sites [23,36].

Epitope prediction was carried out for the HC and HAM/TSP consensus sequences to 27 HLA-I (HLA A1101, HLA A26, HLA B1510, HLA B4402, HLA A01, HLA A0201, HLA A2402, HLA A5101, HLA A03, HLA B2705, HLA A6801, HLA B08, HLA B0702, HLA B2709, HLA B 1402, HLA B1501, HLA B18, HLA B37, HLA B3801, HLAB 3901, HLA B 3902, HLA B4001, HLA B4101, HLA B 4501, HLA B 4701, HLA B 4901, HLA B5101) and 6 HLA-II alleles (HLA DRB1 0101, HLA DRB1 0401, HLA DRB1 0301, HLA DRB1 1501, HLA DRB1 0701, HLA DRB1 1101), using the online bioinformatics tool SYFPEITHI (http://www.syfpeithi.de/Scripts/MHCServer.dll/Epitope Prediction.htm) [37]. This tool uses an algorithm that can predict sequences that have the potential ability to bind to one or more different HLA-I and HLA-II molecules. It also provides information about the epitope sequence, the specificity to the HLA molecule and the HLA binding score for each epitope.

To investigate the possible influence of the described mutations in the gp46 sequences, physico-chemical analysis was performed using Network Protein Sequence Analysis (NPSA) (http://npsa-pbil.ibcp.fr/) [38-42] and the potential protein domain analysis using the GeneDoc software and the Prosite tool, as previously described [43].

Finally, the SWISS-MODEL online tool (http://swissmodel.expasy.org/) [44] was used as a fully automated protein structure homology-modeling server, to infer the possible influence of the amino acid changes at protein secondary structure.

All *env* nucleotide sequences previously deposited in the GenBank and used in the study are listed below with their corresponding accession number: [L26585, L26586, L33265, L33266, AF091494-AF091500, AF092065, L76041-L76049, L76052-L76054, L76056, L76058, L76060, DQ007189-DQ007209, HM770426-HM770440, U81865-U81869, AF077209].

## Competing interest

The authors declare they have no competing interests.

## Authors’ contributions

ACAMM carried out the molecular genetic studies, the bioinformatics analyses and drafted the manuscript. FKB participated in the molecular genetic studies and the bioinformatics analyses. MFCA participated in the cloning. EB participated in the cloning and molecular studies. JPMC had performed the English review and helped to draft the manuscript. LFV contributed to the analysis of the results and helped to draft the manuscript. BGCF contributed with the blood samples and clinical data from HTLV-1 infected individuals. LCJA conceived of the study, participated in its design and coordination and helped to draft the manuscript. All authors read and approved the final manuscript.

## Supplementary Material

Additional file 1 Brief description of previously published domains in the HTLV-1 gp46 protein and their exact aminoacid location. Click here for file
