# Supplementary material for: Molecular characterization of HTLV-1 gp46 glycoprotein from health carriers and HAM/TSP infected individuals
Source: Virol J. 2013 Mar 6;10:75. doi: 10.1186/1743-422X-10-75 (PMC3599561; doi:10.1186/1743-422X-10-75)
Supplement: Additional file 1 — Brief description of previously published domains in the HTLV-1 gp46 protein and their exact aminoacid location. [file 1743-422X-10-75-S1.doc]

| **DOMAIN/SITE** | **DESCRIPTION** | **REFERENCE** |
| --- | --- | --- |
| 197-205aa | Functional domain | Sagara *et al.*, 1996 |
| 75-101aa | Functional domain | Rosenberg *et al.*, 1998 |
| 181-208aa | Functional domain | Rosenberg *et al.*, 1998 |
| 197aa | Mutations at this site are associated to a decrease in cellular fusion. | Rosenberg *et al.*, 1998 |
| 90aa | Mutations around at this site make syncytium and virus entry difficult. | Rosenberg *et al.*, 1998 |
| I173V and A187T | Mutations associated to the induction of neutralizing antibodies | Blanchard *et al.*, 1999 |
| 53-75aa | Region that is characterized by a prevalence of linear epitopes. | Tallet *et al.*, 2001 |
| 86-107aa | Region that is characterized by a prevalence of linear epitopes. | Tallet *et al.*, 2001 |
| 175-209aa | Region that is characterized by a prevalence of linear epitopes. | Tallet *et al.*, 2001 |
| 288-317aa | Region that is characterized by a prevalence of linear epitopes. | Tallet *et al.*, 2001 |
| 90-94aa | Amino acids associated to the interaction with neurophilin 1. | Lambert *et al.*, 2009 |
| 290-312aa | Important region for the binding to Heparan Sulfate Proteoglycans. | Jones *et al.*, 2006 |
| 25-190aa | Receptor Binding Domain (RBD) | Kim *et al.*, 2000 and Manel *et al.*, 2003 |
| D106 and Y114 | Critical residues for the interaction of gp46 to the GLUT-1. | Manel *et al.*, 2003 an Ghez *et al.*, 2010 |
| 80-83aa | YSLY- Addressing motif of gp46 for the plasma membrane. | Ilinskaya *et al.*, 2010 |
